# Mechanism of non-appearance of hiatus in Tibetan Plateau

**DOI:** 10.1038/s41598-017-04615-7

**Published:** 2017-06-30

**Authors:** Jieru Ma, Xiaodan Guan, Ruixia Guo, Zewen Gan, Yongkun Xie

**Affiliations:** Key Laboratory for Semi-Arid Climate Change of the Ministry of Education, College of Atmospheric Sciences, Lanzhou University, Lanzhou, 730000 China

## Abstract

In the recent decade, hiatus is the hottest issue in the community of climate change. As the area of great importance, the Tibetan Plateau (TP), however, did not appear to have any warming stoppage in the hiatus period. In fact, the TP showed a continuous warming in the recent decade. To explore why the TP did not show hiatus, we divide the surface air temperature into dynamically-induced temperature (DIT) and radiatively-forced temperature (RFT) by applying the dynamical adjustment method. Our results show that DIT displayed a relatively uniform warming background in the TP, with no obvious correlations with dynamic factors. Meanwhile, as the major contribution to warming, the RFT effect over the TP played the dominant role. The warming role is illustrated using the temperature change between perturbed and control simulation responses to CO_2_ or black carbon (BC) forcing via Community Earth System Model (CESM). It shows that an obvious warming in the TP is induced by the CO_2_ warming effect, and BC exhibits an amplifying effect on the warming. Therefore, the continuous warming in the TP was a result of uniform DIT warming over a large scale and enhanced RFT warming at a regional scale.

## Introduction

As the Earth’s “third pole”, the Tibetan Plateau (TP) is one of the most important areas for global weather and climate change. It has the highest altitude and most complex terrain structure in the world. The dynamic and thermal effects of the TP on the atmospheric circulation and Asian monsoon in China, East Asia and even the world are huge^1^. The sensible heating on the surface of the TP in summer can drive low-level water vapor to converge rapidly, and to generate convective precipitation and produce latent heating over the TP^2, 3^. More importantly, the thermal forcing of the TP interacts with that of the Iranian Plateau (IP) and other surrounding continents, thereby affecting atmospheric vertical motion and circulation^4^. The thermal forcing of the Tibetan-Iranian-Plateau sensible-heat-driven air pump has a dominant influence on moist convection and the continental summer monsoon; its thermal status in the TP can influence global climate, particularly the Asian monsoon^5^. Wu *et al*.^6^ showed that orographic and thermal forcing in the TP acts to enhance the coupling between the lower-tropospheric and upper-tropospheric circulations and the coupling between the subtropical and tropical monsoon circulations. The weakening of sensible and latent heating over the TP in summer results in reduced thermal forcing to the atmosphere, and the coupling between the tropical and subtropical climates is relaxed to some extent. Thus, the anomalous pattern with more rain in the south and less in the north is formed^7^. Furthermore, the TP is a sensitive area to global climate change, which influences the variability of hydrosphere, cryosphere, ecosystem, living condition, and socio-economic development within the plateau^8, 9^. Some research has shown that the air temperature increased continuously over the TP since the mid-1950s based on measurements from meteorological stations, particularly in winter^10–12^, which affected rainfall, lakes, regional glaciers, normalized difference vegetation index (NDVI), and aridity variation^13–17^. Temperature spatiotemporal evolution over the TP and its dynamic and thermal effects on climate change are particularly important^18, 19^.

The global mean surface temperature warming trend slow-down, referred to as global warming hiatus, started around 2000 and has caused many concerns and stimulated discussion^20^. The warming hiatus shows large regional differences in terms of temperature change. There are two major explanations of hiatus, a stratospheric water-vapour increase or solar variability results in radiative forcing reduction^21^, and oceanic internal climate variability, including natural variability influenced by a La Niña-like cooling in the tropical Pacific^22^ and heat transported to deeper layers of the ocean^23^. However, several studies noted the cooling effect over land during the hiatus period^24^. Huang *et al*.^25^ proposed a possible dynamic mechanism for the cooling over the Eurasia and North American continents, and pointed out that the downward decadal modulated oscillation (DMO) excited by internal climate variability and the Arctic Amplification might have reduced the radiatively-forced warming, and caused the recent global warming slow-down and cooling over the Northern Hemisphere (NH) land. Guan *et al*.^26^ pointed out that dynamically-induced cooling effect offset radiatively-forced warming effect and resulted in warming hiatus in the NH. But, some research found an accelerated warming trend over the TP in contrast to the global warming hiatus during 1998–2013^27^, and these authors noted that recent accelerated warming might be attributable to cloud-radiation feedback from the thermal viewpoint. The temperature increase and surface heat flux changes over the TP have been analyzed in many studies^28, 29^. However, the cause for the continuous warming over the TP in the recent hiatus period has not been identified.

In this study, the mechanism for the continuous warming over the TP during 1980–2012 and recent hiatus period is investigated using a recently developed method. For the dynamical adjustment method, the surface air temperature (SAT) is divided into dynamically-induced temperature (DIT) and radiatively-forced temperature (RFT). In section 2, we explore the causes for the continuous warming trend over the TP while there was a cooling trend in the other areas, and analyze the characteristics and roles of DIT and RFT variation over the TP. Additionally, we try to explain main causes of DIT and RFT variability in the TP. Summary and discussion are presented in section 3. Details of the datasets and method used are given in section 4.

## Results

**Figure 1:**
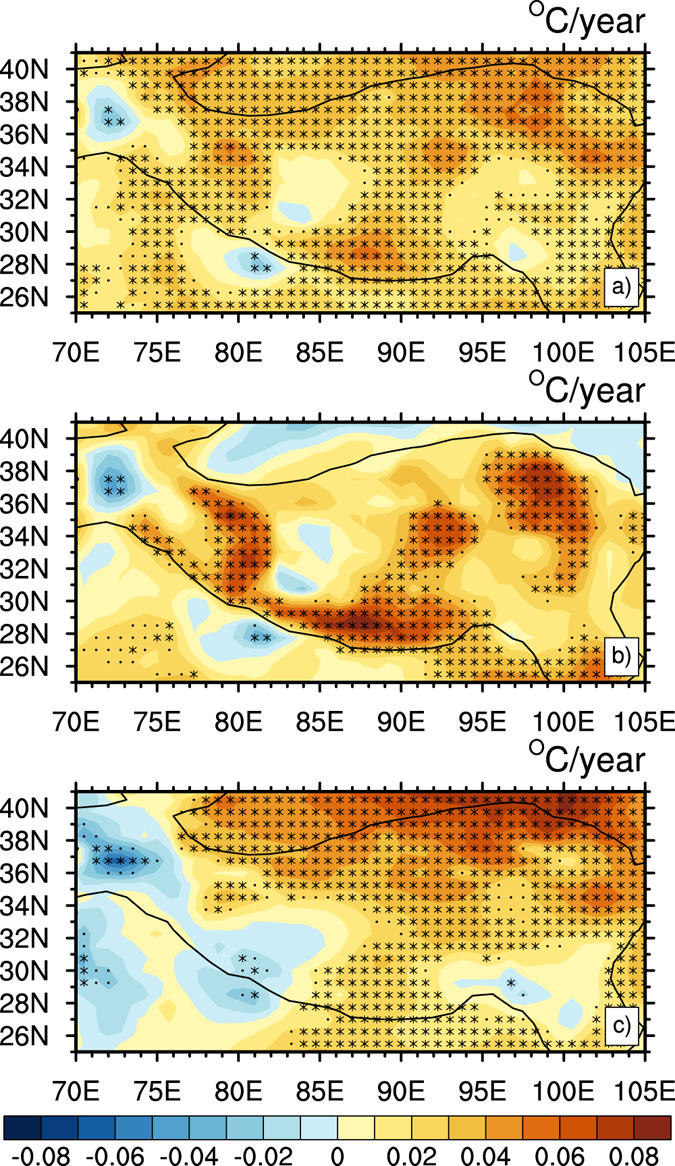
Distribution of temperature trend (°C/year) over the TP during 1980–2012: (**a**) annual mean, (**b**) winter mean and (**c**) summer mean. Asterisk and black dot indicate the trends are significant at 95% and 90% confidence levels according to a two-tailed Student’s *t*-test, respectively. Black contour indicates where the elevation equals 1800 m, consistent with Wu *et al*.^8^. Figure 1 is generated using NCL version 6.3.0, open source software free to public, by UCAR/NCAR/CISL/TDD, http://dx.doi.org/10.5065/D6WD3XH5.

**Figure 2:**
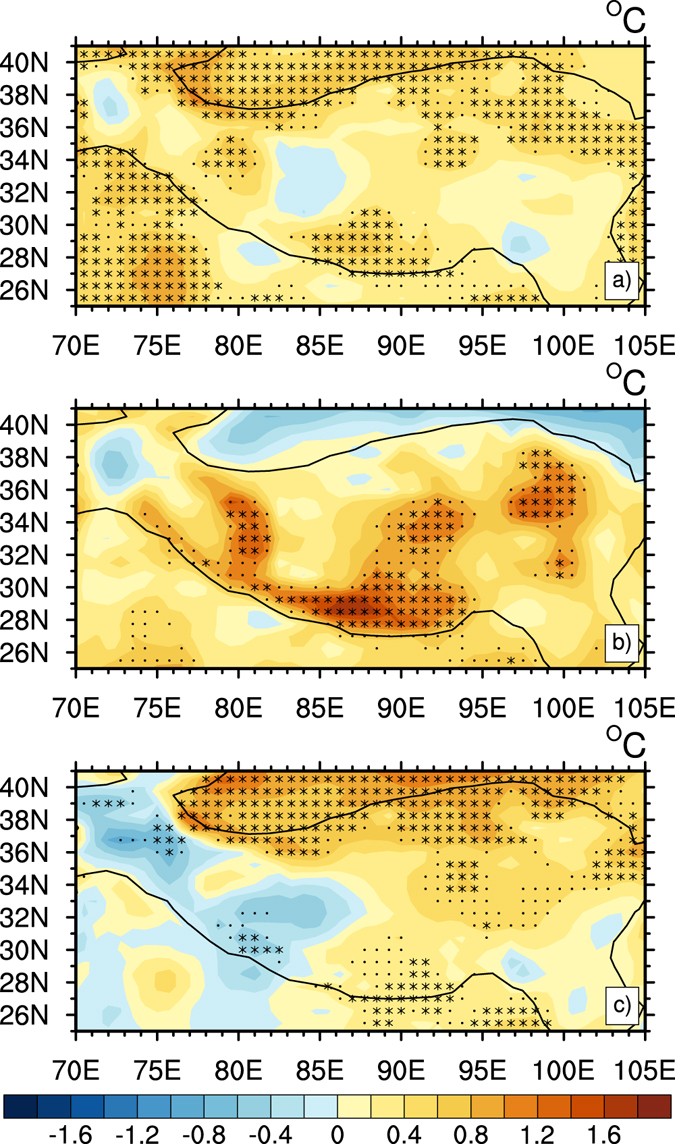
Same as Fig. 1, except for decadal temperature difference (°C) over the TP (2000–2009 minus 1990–1999). Figure 2 is generated using NCL version 6.3.0, open source software free to public, by UCAR/NCAR/CISL/TDD, http://dx.doi.org/10.5065/D6WD3XH5.

To understand the evolution of SAT in the whole TP, the spatial distributions of linear trend of SAT for annual mean, winter (December to February) mean and summer (June to August) mean are shown in Fig. 1, which shows a good agreement with the figure of station observations (see Supplementary Fig. S1). In the past 30 years, the annual mean SAT in the TP (Fig. 1a) presented an overall upward trend, except for a small area in the southwest of the plateau. Using the confidence level of 95%, large values of warming appeared in the south of the Kunlun Mountains, the middle east of the Himalaya Range, the east of the Hoh Xil Range and the Qaidam Basin to Qinghai Lake, and a slight cooling trend appeared in the south of the Himalaya. Compared to the annual mean SAT (Fig. 1a), the warming trend of SAT in winter (Fig. 1b) was more significant; the southwestern and northern parts of the Himalaya Range showed a small cooling trend, and the rest showed a warming trend. Similar to Fig. 1a, a significant increasing trend appeared in the Kunlun Mountains to the Himalaya Range, west of the Qinghai Plateau, and east of the Qaidam Basin, all passing the confidence level of 95%. The trend in summer (Fig. 1c) was obviously different from the trend in annual mean (Fig. 1a) and winter mean (Fig. 1b). Most areas of the plateau showed a warming trend, and the high value centers were over the north of the plateau, also significant at the 95% confidence level. Therefore, the warming in the TP was obvious in winter. Previous results^27, 30^ reported an enhanced warming in the TP during the past decades. Duan and Xiao^27^ proposed that rapid climate warming persisted over the TP during 1998–2013, which is different from warming hiatus in the rest of East Asia. To check whether the hiatus appeared in the TP, we calculated the decadal differences of SAT for annual, winter and summer mean (Fig. 2). The decadal difference of annual SAT (Fig. 2a) showed a continued warming almost over the entire TP in the hiatus decade compared to the previous decade. The difference of winter SAT (Fig. 2b) exhibited a significant warming over the northwest, middle and northeast of the plateau. Figure 2c shows difference in summer SAT, with warming areas in the northern and southern parts of the TP, whereas cooling centers occurred in the western and small eastern parts of the plateau. Figure 2 displays a similar pattern in the linear trend of mean SAT (Fig. 1), which indicates a continuous warming of SAT in the TP during the hiatus decade, being warmer in winter, which is consistent with other recent studies^27^.

**Figure 3:**
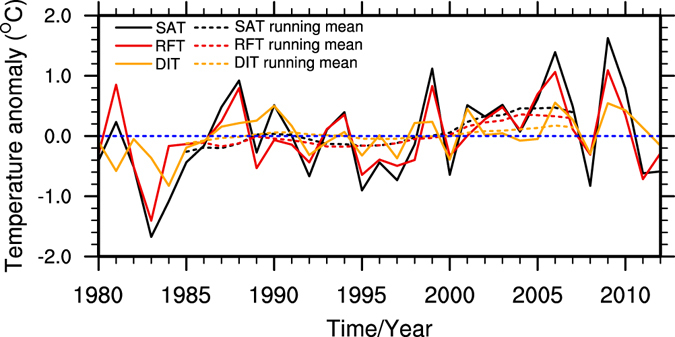
Time series of decomposed winter temperature (°C) of the TP. Solid lines represent the time series of the original data, and dashed lines are the 11-year running mean. Black lines for SAT; red lines for RFT; and orange lines for DIT. Figure 3 is generated using NCL version 6.3.0, open source software free to public, by UCAR/NCAR/CISL/TDD, http://dx.doi.org/10.5065/D6WD3XH5.

**Figure 4:**
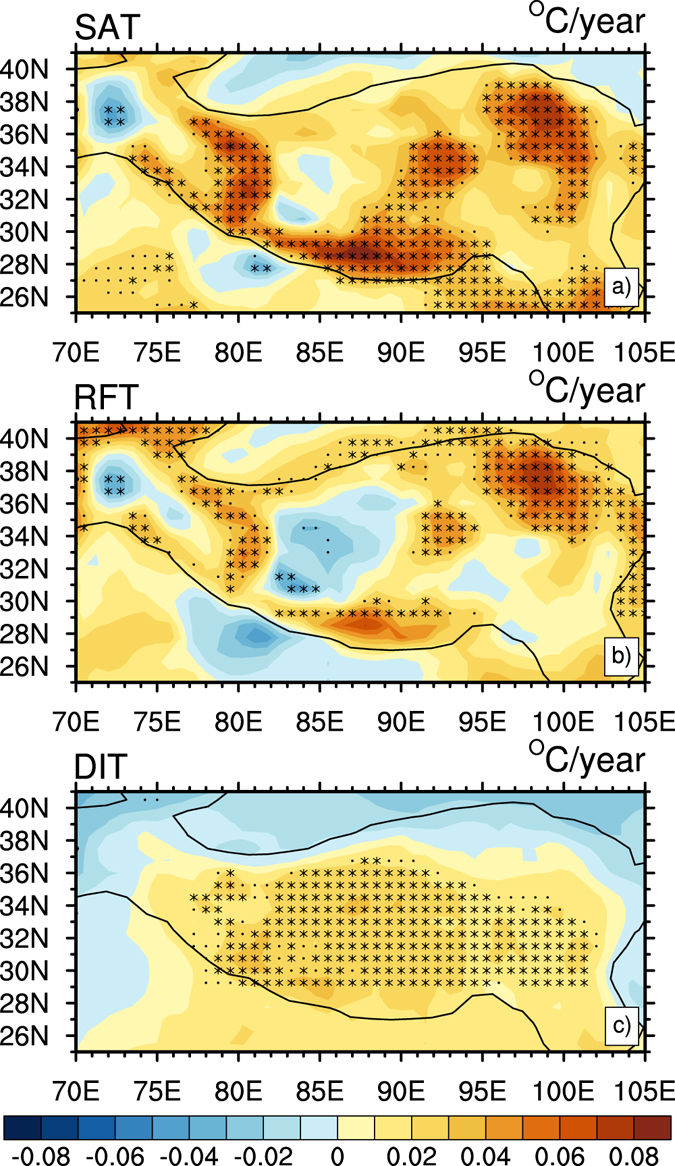
Trends of decomposed winter temperature over the TP during 1980–2012 (°C/year): (**a**) SAT, (**b**) RFT and (**c**) DIT. Asterisk and black dot indicate the trends are significant at 95% and 90% confidence levels according to a two-tailed Student’s *t*-test, respectively. Figure 4 is generated using NCL version 6.3.0, open source software free to public, by UCAR/NCAR/CISL/TDD, http://dx.doi.org/10.5065/D6WD3XH5.

As noted earlier, the temperature in the TP increased continually and significantly in winter, which was different with the warming stoppage appeared in other regions^25^. To explore the causes for the continuous warming in the TP, we decomposed the winter SAT into two parts by applying the dynamical adjustment method, and named them DIT and RFT, respectively. Figure 3 shows the time series of SAT, RFT and DIT in winter during 1980–2012. The SAT time series in winter (black lines) showed obvious inter-decadal variability. There were two high peaks of warming rate in the past 30 years: one around 1988 and the other around 2007. The first warming peak was in the enhanced warming period of the 1980s, but the second higher warming peak was in the hiatus period that was always considered as a cooling period in mid-high latitude of Eurasia and Northwest of North America^26^. Although the SAT had a cooling trend since 2006, it showed a warming trend in the entire period of 1980–2012. The warming rate change over the TP had a continuous increasing trend after 2000. Compared with the previous decade, the warming rate seemed stronger during 2000–2012 than the previous decade in Fig. 3. The variability of winter RFT (red lines) are similar to that of winter SAT (black lines), with an upward trend, but the trend of winter DIT (orange lines) was different and showed a gradual increase since about 1995, suggesting that DIT had a warming effect on SAT for nearly 20 years. The warming in the TP was the result of both RFT and DIT in the past 30 years. The spatial distributions of linear trend of SAT, RFT and DIT in winter are illustrated in Fig. 4. We can see that the RFT (Fig. 4b) had a similar spatial pattern as SAT (Fig. 4a), but the strength of the trend was weaker than that of SAT. The maximum values of warming were about 0.07 °C/year and 0.05 °C/year in SAT and RFT, respectively. The centers of warming in RFT appeared in the Himalaya Range, east of the Hoh Xil Range, and the Qilian Mountains, passing the confidence level of 95%. DIT (Fig. 4c) showed a slightly warming trend almost in the whole TP for the confidence level of 95%. Distribution of increasing trend in DIT was uniform, and the maximum of warming trend was about 0.03 °C/year.

**Figure 5:**
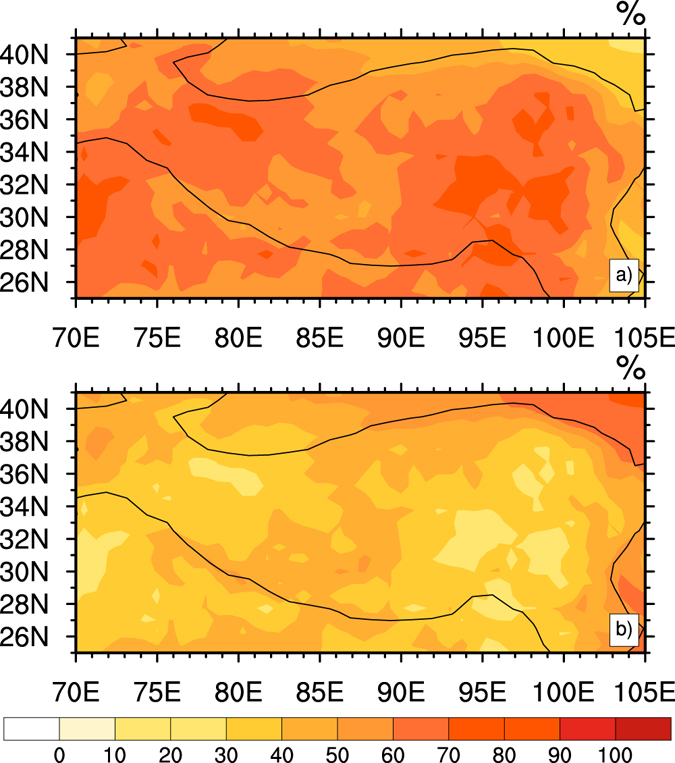
Contributions of RFT (**a**) and DIT (**b**) to SAT over the TP from 1980 to 2012 (%). Figure 5 is generated using NCL version 6.3.0, open source software free to public, by UCAR/NCAR/CISL/TDD, http://dx.doi.org/10.5065/D6WD3XH5.

To quantify the roles of radiative and dynamic parts in the SAT warming over the TP, we calculated the contributions of RFT and DIT to SAT in winter over the TP during the period of 1980–2012 (Fig. 5). Basically, the contribution from RFT (Fig. 5a) was much larger than that from DIT (Fig. 5b). The spatial distribution of RFT contribution (Fig. 5a) had high values in the middle east, southwest and northwest of the TP, and the high values of DIT contribution (Fig. 5b) were located in the middle west and southeast of the TP. As for regional averages in Fig. 5, RFT and DIT contributed 60.9% and 39.1% to the SAT over the whole TP, respectively. The contributions are consistent with the results from Fig. 3, which the relative contributions of RFT and DIT to SAT are 66.9% and 33.1%, respectively. This further illustrates that SAT was mostly determined by RFT, and that dominant RFT had a warming effect on SAT in the TP. We also calculated the decadal differences in winter for SAT, RFT and DIT between 1990–1999 and 2000–2009 (see Supplementary Fig. S2). The SAT decadal difference between the hiatus decade and the previous decade displayed a significant warming center with significance of 95% over the Himalaya, the western Kunlun Mountains, the eastern Hoh Xil Range, and south of Qinghai Lake. A similar pattern can be found in the RFT decadal difference, but the range of difference in RFT was smaller. The DIT decadal difference exhibits a uniform increase in a large area over the TP during the hiatus, but the difference was obviously smaller than that in SAT and RFT. This further verifies the RFT change dominated the warming continually in the TP during the hiatus decade, and the uniform warming of DIT had a slightly increasing contribution to the SAT.

**Figure 6:**
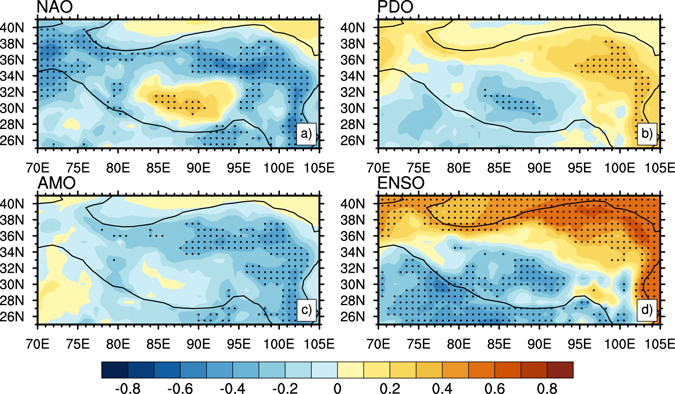
Correlation coefficients between DIT and dynamic factors in winter: (**a**) NAO with DIT, (**b**) PDO with DIT, (**c**) AMO with DIT, and (**d**) ENSO with DIT. Black dot indicates a 90% confidence level according to a two-tailed Student’s *t*-test. Figure 6 is generated using NCL version 6.3.0, open source software free to public, by UCAR/NCAR/CISL/TDD, http://dx.doi.org/10.5065/D6WD3XH5.

As illustrated earlier, different contributions of RFT and DIT to warming in the TP were dominated by different factors. For example, the variation of DIT was closely associated with the dynamic factors^31^. The North Atlantic Oscillation (NAO), Pacific Decadal Oscillation (PDO), Atlantic Multidecadal Oscillation (AMO), and El Niño-Southern Oscillation (ENSO) as the most typical dynamic factors have played major roles in the climate change^32^. Goswami *et al*.^33^ proposed that the strong NAO influences climate change by producing the tropospheric anomaly of meridional temperature gradient over Eurasia. The PDO can modulate the interannual relationship between ENSO and global climate. In the positive PDO phase, the warm and wet conditions are transported into the Asian continent through the southerly wind anomalies along the western flank of the anomalous high pressure^34, 35^. Temperature over the TP can increase quickly when the AMO enters its warm phase, which further influences the monsoon by strengthening or weakening the meridional temperature gradient between TP and the tropical Indian Ocean in the lower and mid troposphere^36^. The ENSO signal can be further transported outside the tropical region by the Hadley circulation and teleconnection, thus affecting Asian temperature change^25, 37^. As the indices of internal climate variability, the combined effect of NAO, PDO, AMO and ENSO on inter-decadal, decadal and interannual time scales may affect atmospheric vertical and horizontal motions over the TP, through changing the asymmetric meridional and zonal temperature gradients and atmospheric circulation anomalies between the TP and different oceanic regions, thereby affecting local DIT warming and climate change over the TP. The correlations between DIT and each of NAO, PDO, AMO, and ENSO indices from 1980 to 2012 are shown in Fig. 6. Using the confidence level of 90%, Fig. 6a indicates that the NAO was negatively correlated with DIT in the TP except for the central plateau, and a negative correlation was obvious in the Southern Qinghai Plateau. This illustrates that the NAO had a cooling effect on DIT in the northwestern and eastern parts of the TP. Figure 6b displays that the PDO was negatively correlated with DIT southwest of the TP and positively correlated with DIT northeast of the TP, which suggests the PDO had a cooling effect on DIT southwest of the TP and a warming effect on DIT northeast of the TP. The AMO (Fig. 6c) was negatively correlated with DIT in the northeastern plateau, which suggests the AMO had a cooling effect on DIT in that area. Finally, the spatial distribution of correlation between ENSO and DIT (Fig. 6d) was similar to that between PDO and DIT (Fig. 6b), namely the ENSO was negatively correlated with DIT southwest of the TP and was positively correlated with DIT northeast of the TP. As the occurrence of PDO had a close relationship with ENSO, they had similar effects. It showed that the ENSO not only had a cooling effect on DIT in the southwestern TP but also had a warming effect on DIT in the northeastern TP. The comparison in Fig. 6a–d illustrates that the NAO, PDO, AMO, and ENSO had close relationship with DIT and had relatively uniform warming effects on DIT in the whole TP during 1980–2012.

**Figure 7:**
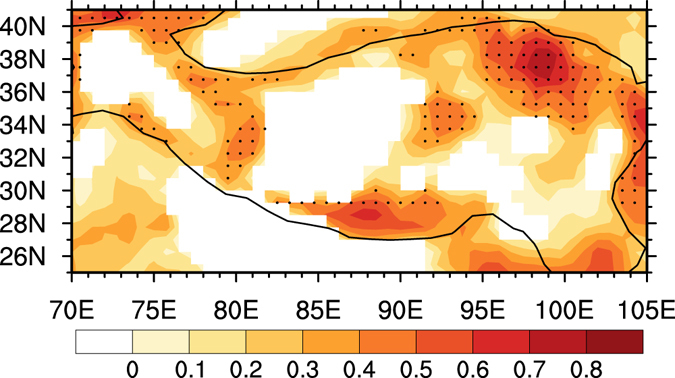
Correlation coefficient between RFT and CO_2_ in winter. Black dot indicates a 95% confidence level according to a two-tailed Student’s *t*-test. Figure 7 is generated using NCL version 6.3.0, open source software free to public, by UCAR/NCAR/CISL/TDD, http://dx.doi.org/10.5065/D6WD3XH5.

Different from the small contribution of DIT to SAT in the TP, the RFT had a dominant role in the continuous warming in the TP. Several studies indicated that the role of radiative forcing was critical in the warming over East Asia, and that radiative factors had major roles especially in enhanced warming over the semi-arid regions^32^. The variation of RFT was considered closely related to the changes in greenhouse gases, land cover, human activity, cloud-radiation, aerosol, vegetation index, and other factors^38, 39^. Carbon dioxide (CO_2_), as one of the important greenhouse gases, has increased continuously since the industrial revolution. Global-mean atmospheric CO_2_ concentration has increased year by year due to human activities, and reached 396.0 parts per million (ppm) in 2013^40^. The relationship between RFT and atmospheric CO_2_ concentration was calculated and shown in Fig. 7, which shows RFT has a high correlation with CO_2_ over the western, central, southern, and northeastern TP. Figure 7 suggests the increased CO_2_ emission may has a major contribution to the RFT warming over the TP. Our results further verify the dominant role of CO_2_ in RFT increase over the TP, which agrees with the results of numerical experiments^41^.

**Figure 8:**
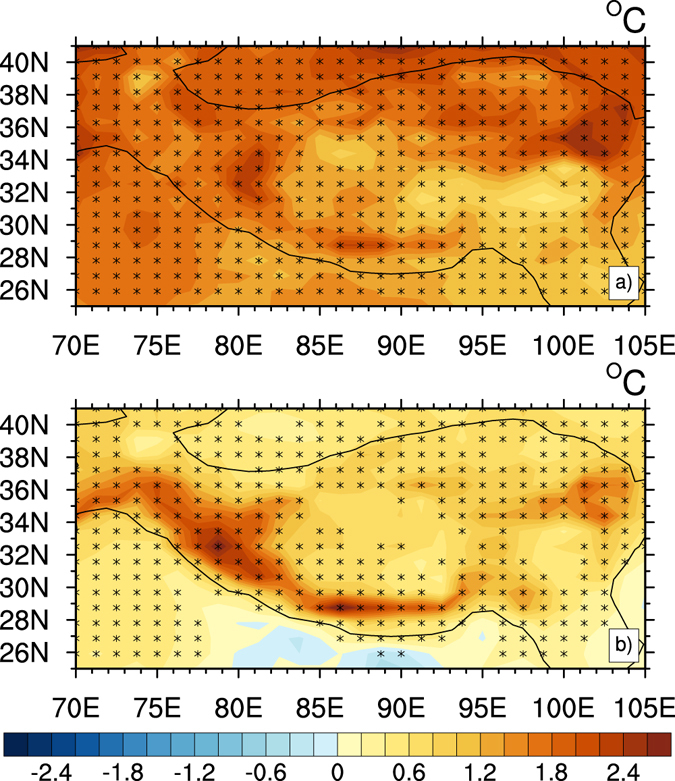
Difference of mean temperature in 60 years (°C) between perturbed and control simulation response to CO_2_ and BC: (**a**) Temperature difference due to CO_2_ forcing, and (**b**) temperature difference due to BC forcing. ﻿Asterisk dot indicates a 95% confidence level according to a two-tailed Student’s t-test. ﻿Figure 8 is generated using NCL version 6.3.0, open source software free to public, by UCAR/NCAR/CISL/TDD, http://dx.doi.org/10.5065/D6WD3XH5.

In addition to CO_2_, increased black carbon (BC) aerosol emission is also considered as one of the major factors causing the warming over the TP^42^. Numerical simulation results showed that BC heating effect can enhance and accelerate snow melting in the TP and Himalayas^43^. The TP is located in the vicinity of densely industrialized regions, in the middle of two largest BC aerosol generating countries–India and China^44^. BC emission was increasing rapidly from 2000 to 2010 in both India and China, and the growth trend of BC emission is more significant in India^45^. BC aerosol directly heats the atmosphere, makes the snow surface dark, which decreases albedo and causes positive snow-albedo feedback. BC aerosol could be transported to the TP and deposited on the surface of ice and snow by westerly wind and monsoon, furthermore reducing snow albedo and accelerating the melting of snow, thus affecting land-atmosphere interaction and climate change over the TP^46, 47^. Therefore, these processes further affect the temperature variation over the TP, especially in its high-elevation regions. Using a state-of-the-art global climate model, Xu *et al*.^48^ confirmed that BC contributed to the snow retreat trend, and explained the roles of various anthropogenic factors in temperature and cryosphere changes over the Himalaya. Figure 8 shows the simulated difference of mean temperature in 60 years between perturbed and control simulation responses to CO_2_ and BC. CO_2_-induced temperature change pattern (Fig. 8a) shows an overall increase, an amplified warming concentrated in the Himalaya, west of the Kunlun Mountains, and from Lake Qinghai to the Qilian Mountains. Figure 8a indicates that CO_2_ forcing mainly contributed to the warming in the middle west and northeast of the plateau, and induced large-scale warming effect. Compared with CO_2_ radiative forcing, the temperature change induced by BC forcing (Fig. 8b) was obviously less. However, the high value centers in BC-induced warming pattern were more concentrated over the Himalaya Range and northeast of the Bayan Har Mountains, because the warming amplitude induced by BC forcing was greater in these mountainous areas. BC-induced warming pattern (Fig. 8b) suggests that increased BC emission was one of the major causes for RFT warming, which mainly amplified warming in the Himalaya and northeast of the Bayan Har Mountains. Therefore, Figs. 7 and 8a illustrate that CO_2_ dominated the RFT warming over the TP, and some regions including the Himalaya, Qinghai Lake, and south of the Qilian Mountains were more sensitive to CO_2_ forcing. Figure 8b indicates BC emission mainly amplified the warming at the high elevation, especially in the Himalaya Range and northeast of the Bayan Har Mountains.

## Summary and Discussion

In this paper, we analyzed possible causes for continuous warming over the TP under the global warming hiatus by using the dynamical adjustment method and simulated experiments. The SAT increased in most areas over the TP during the period of 1980–2012, particularly in winter. Moreover, the variation of RFT had similar characteristics as SAT during 1980–2012, but DIT showed a uniform warming trend over the whole TP. For the regional average, RFT and DIT changes contributed 60.9% and 39.1% to SAT variation over the whole TP, respectively. This indicates that warming induced by RFT was more significant; so, RFT was the major reason and DIT was the secondary reason for the continuous warming over the TP. The NAO, PDO, AMO, and ENSO had close relations with the variation of DIT, and induced overall uniform warming effects on DIT variation over the whole TP. As the main greenhouse gas, CO_2_ played a large warming role in RFT change over the TP. BC aerosol further amplified warming via direct heating of the atmosphere and causing positive snow-albedo feedback in the Himalaya Range and northeast of the Bayan Har Mountains. Our results are robust in showing the dominant effect of RFT variability on warming in the TP. Therefore, further investigation should focus on radiatively-forced processes, and on possible radiatively-forced factors that caused the enhanced increasing of RFT over the TP. Also, the implementation of proper BC aerosol parameterizations and improvement of the parameters associated with BC aerosol in the model may lead to a better description of climate warming and its vital effects on ecosystems over the TP.

Besides BC aerosol and CO_2_ emission, other factors have been suggested for contributing to the enhanced warming in the TP. Temperature variation depends on elevation, and its horizontal pattern is coincided with that of glacier change^49, 50^, in which the positive snow/ice-albedo feedback with reduced surface snow/ice and increased net absorption of solar radiation strengthen the warming over high-elevation regions in the TP^51^. An increase in downward longwave radiation in response to increase in water vapour content in the atmosphere at higher elevation may contribute to enhanced warming in the TP^52^. In addition, other environmental elements including cloud amount, atmospheric circulation, specific humidity, vegetation cover type, and land use change are also relevant factors for the recent climate warming over the TP^30, 53^. Therefore, further investigation to quantify the impacts of these factors on the warming over the TP is needed.

## Methodology

The data of monthly surface air temperature at 2 m from 1979 to 2012 from the European Centre for Medium-Range Weather Forecasts (ECMWF) are used in this study. They are the third-generation reanalysis data of the ERA-Interim, and are globally gridded at 0.75° × 0.75° resolution. The data quality in the TP has been checked in previous studies, and the data is considered as one of the best datasets for studying the TP^54–56^. ERA-Interim is a global atmospheric reanalysis starting from 1979, and is continuously updated in real time. The data assimilation used in ERA-Interim is 4-dimensional variational analysis (4DVar) with a 12-hour analysis window, which can reproduce the basic characteristics of surface air temperature and describe the seasonal and inter-decadal variation of surface air temperature in the TP^55^. The ERA-Interim data are available for download: (http://apps.ecmwf.int/datasets/data/interim-full-daily/levtype=sfc/).

The indices of NAO^57^, PDO^58^, AMO^59^, and ENSO^60^ during 1980–2011 are downloaded from the Royal Netherlands Meteorological Institute (KNMI) Climate Explorer (http://climexp.knmi.nl). The observational sea level pressure (SLP) data used to separate SAT are from the National Oceanic and Atmospheric Administration (http://www.esrl.noaa.gov/psd/data/gridded/data.20thC_ReanV2.monolevel.mm.html). In addition, the historical CO_2_ concentration during 1980–2005 and predicted CO_2_ concentration during 2006–2012 under the Representative Concentration Pathways 8.5 (RCP 8.5) scenario are used to understand the effect of CO_2_ on the warming over the TP; these CO_2_ concentration data are available from http://tntcat.iiasa.ac.at:8787/RcpDb/dsd?Action=htmlpage&page.

The 75-year temperature field from a perturbed simulation with present-day BC or CO_2_ forcing and a long-term control simulation with preindustrial BC or CO_2_ forcing in a coupled model by Xu *et al*.^48^ were used in our study. The temperature differences between the last 60 years of the perturbed and long-term control simulations were calculated. Xu *et al*.^48^ used a high-resolution ocean-atmosphere global climate model (Community Earth System Model) and observationally constrained BC aerosol forcing to investigate the role of BC in cryosphere change over the Himalaya. BC radiative forcing was constrained by multiple sources of observations to calculate its direct radiative forcing, and the concentration of CO_2_ was calculated offline and then prescribed to the model. More detailed information for the calculation can be found in Xu *et al*.^48^.

The dynamical adjustment method is based on the partial least-squares (PLS) regression of SAT against SLP^61, 62^. The PLS regression predicts a dependent variable (predictand) based on a set of independent variables (predictors), and has been applied in various climate research^63, 64^. The adjustment process removes most of the variability that is induced by variation in the atmospheric circulation, and can be used for climate diagnostic studies of both short-term climate fluctuations and long-term trend. By applying the dynamical adjustment method, raw SAT is separated into DIT and RFT. The DIT variability is related to atmospheric circulation variation, and the other part (RFT) is related to radiatively forced processes, such as greenhouse gas accumulation, aerosol emission, stratospheric ozone depletion, volcanic eruption, and local anthropogenic forcing. This methodology in this study is used to explore the possible causes for warming over the TP under the global warming hiatus. In detail, both temperature and SLP were standardized, and temperature time series is high-pass filtered to prevent the overfitting first. Then, a one-point cross-correlation map between the temperature time series at each point and SLP field north of 20°N was calculated. In the next step, the first PLS predictor time series was obtained by projecting the SLP field onto the correlation pattern and weighing each grid point by the cosine of its latitude. Finally, regressing the PLS predictor to temperature and SLP field so that the first dynamic component DIT1 of DIT and the residual field about temperature or SLP can be calculated. The above processes were repeated and iterated three times in this study, to obtain the dynamic components DIT1, DIT2 and DIT3. So, the DIT associated with changes of atmospheric circulation and the RFT associated with radiatively-forced factors are as follows:

1 $${\rm{DIT}}={\rm{DIT}}1+{\rm{DIT}}2+{\rm{DIT}}3$$

2 $${\rm{RFT}}={\rm{SAT}}-{\rm{DIT}}$$

The method has been successfully used in studying the enhanced warming and hiatus over the mid-to-high latitude of the NH in different periods^26^. Since the TP is a unique area, applying this method to the TP will advance studies of dynamic and thermodynamic effects of the TP.

## Electronic supplementary material

Supplementary Information
