# Supplementary material for: Mechanism of non-appearance of hiatus in Tibetan Plateau
Source: Sci Rep. 2017 Jun 30;7:4421. doi: 10.1038/s41598-017-04615-7 (PMC5493643; doi:10.1038/s41598-017-04615-7)
Supplement: Supplementary file 1 — Supplementary Information [file 41598_2017_4615_MOESM1_ESM.pdf]

Mechanism of non-appearance of hiatus in Tibetan Plateau

Jieru Ma[1], Xiaodan Guan[*], Ruixia Guo[1], Zewen Gan[1] and Yongkun Xie[1]

[1]Key Laboratory for Semi-Arid Climate Change of the Ministry of Education, College of Atmospheric Sciences, Lanzhou University, Lanzhou 730000, China

Corresponding author: Xiaodan Guan

Key Laboratory for Semi-Arid Climate Change of the Ministry of Education
College of Atmospheric Sciences
Lanzhou University
Lanzhou 730000, China
guanxd@lzu.edu.cn

List of supporting figures

Supporting Figures

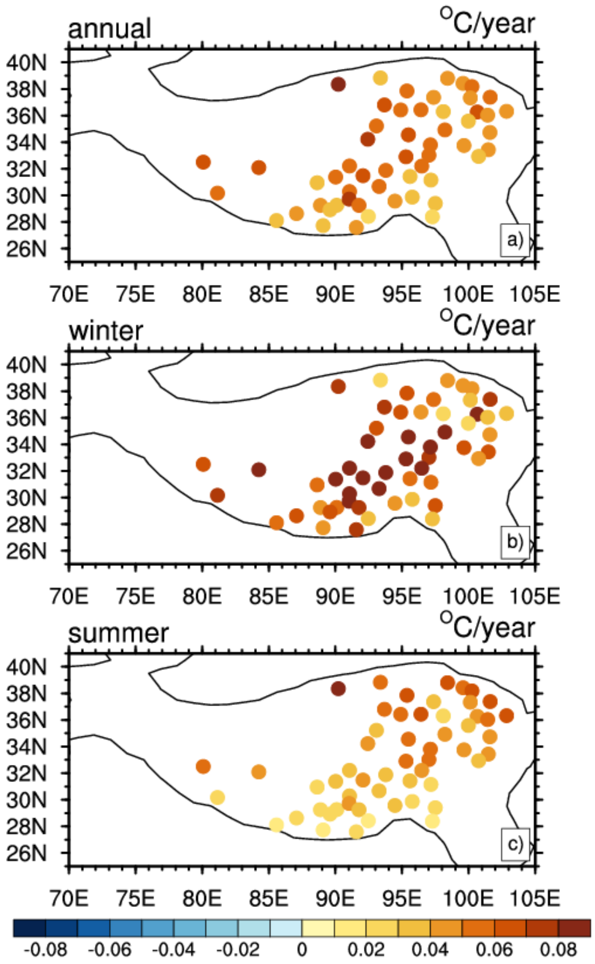

Figure S1 The trend (℃/year) of station temperature in the TP during 1980-2012: (a) annual mean, (b) winter mean and (c) summer mean. Black contour indicates where the elevation equals 1800 m. The monthly mean temperature observations are provided by the China Meteorological Administration (CAM). Figure S1 is generated using NCL version 6.3.0, open source software free to public, by UCAR/NCAR/CISL/TDD, http://dx.doi.org/10.5065/D6WD3XH5.

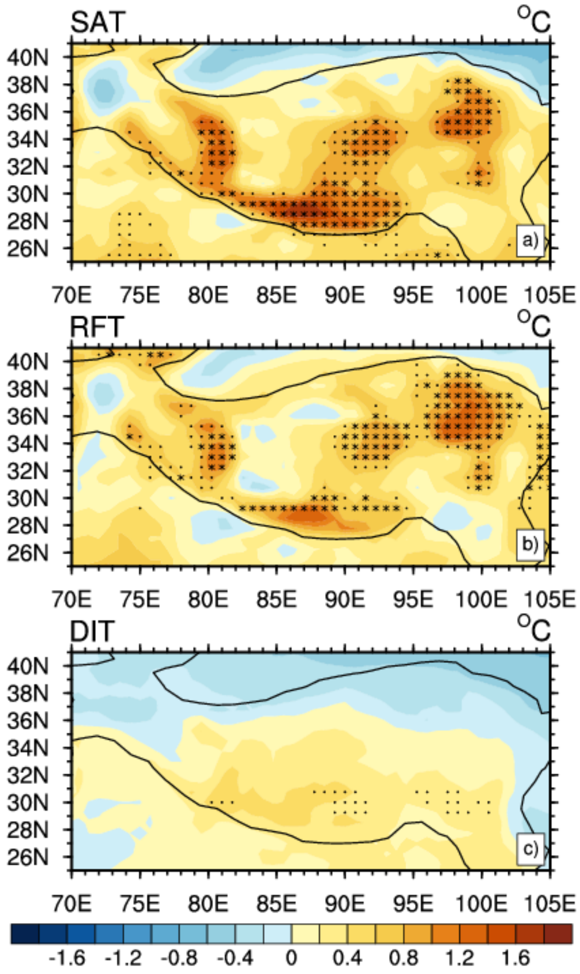

Figure S2 The decadal temperature difference (℃) over the TP (2000-2009 minus 1990-1999) for winter SAT (a), winter RFT (b) and winter DIT (c). Figure S2 is generated using NCL version 6.3.0, open source software free to public, by UCAR/NCAR/CISL/TDD, http://dx.doi.org/10.5065/D6WD3XH5.
